# Aglycosylated Immunoglobulin G1 Fc Stabilized Through Disulfide Bond Addition Exhibits Compositional Homogeneity and Retains Fc γ Receptor IIIa/CD16a Binding

**DOI:** 10.3390/antib15040055

**Published:** 2026-06-25

**Authors:** Anjali Shenoy, Daniel J. Falconer, Adam W. Barb

**Affiliations:** 1 Department of Biochemistry and Molecular Biology, University of Georgia, Athens, GA 30602, USA; 2 Roy J. Carver Department of Biochemistry, Biophysics and Molecular Biology, Iowa State University, Ames, IA 50011, USA; 3 Complex Carbohydrate Research Center, University of Georgia, Athens, GA 30602, USA; 4 Department of Chemistry, University of Georgia, Athens, GA 30602, USA

**Keywords:** N-glycosylation, yeast surface display, glycobiology

## Abstract

Background: The interaction between human immunoglobulin G (IgG)1 Fc and the Fc gamma receptor (FcγR) IIIa/CD16a elicits protective immune responses. Antibody N-glycosylation stabilizes the FcγR-binding interface and is thus essential for interaction with wildtype IgG1 Fc. Furthermore, the N-glycan introduces substantial compositional and functional heterogeneity, with distinct glycoforms providing different affinities and discrete responses in vivo. Accordingly, various engineering endeavors to improve antibody binding strive to boost the therapeutic efficacy of monoclonal antibodies but do not directly address compositional heterogeneity. Objective: Here, we describe a previously unexplored approach to engineer IgG1 Fc. We eliminated carbohydrate heterogeneity by removing the N-glycan but stabilizing the FcγR-binding interface with disulfide bonds. Conclusions: These newly generated Fc domains served as a starting point for protein engineering through yeast surface display to enhance receptor-binding affinity. We recovered Fc variants from this approach that demonstrated FcγRIIIa binding affinities comparable to the starting sequence and thus serve as a proof-of-principle for this strategy.

## 1. Introduction

Monoclonal antibody therapeutics are currently being used to combat various cancer types, infectious diseases, and autoimmune disorders [1,2]. The crystallizable fragment (Fc) of immunoglobulin G1 (IgG1) enhances antibody-mediated protection by binding to various immune effector molecules, including Fc gamma receptor IIIa (FcγRIIIa)/CD16a. FcγRIIIa on natural killer cells elicits antibody-dependent cell-mediated cytotoxicity (ADCC), a protective mechanism that kills diseased or invading cells. As a result, IgG1 Fc has been the subject of various engineering endeavors to modify affinity and selectivity. Successful engineering approaches in this space have the potential to greatly enhance the therapeutic potential of existing drugs by increasing ADCC and other antibody-mediated immune functions, though significant challenges exist [3,4,5,6,7].

The IgG Fc contains a conserved N-glycan that is required for interacting with many key proteins, including the FcγRs [8,9,10]. The Fc N-glycan stabilizes the C’E loop, preorganizing the binding interface and increasing affinity for the FcγRIIIa receptor [11]. Elimination of the Fc N-glycan destabilizes the C’E loop to reduce affinity. Furthermore, the Fc N-glycan composition itself influences FcγRIIIa-binding affinity [12,13]. N-glycan compositional heterogeneity results from the template-independent synthetic pathway, and significant heterogeneity often results from industrial antibody expression platforms [14]. Furthermore, several groups have reported on N-glycan-modifying enzymes capable of mediating transglycosylation reactions, wherein a desired N-glycan from a donor substrate can be attached to an acceptor protein. Multiple versions of these enzymes have been generated and characterized. Each of them demonstrates varying levels of efficacy and N-glycan specificity [15,16,17,18,19]. However, these approaches are unlikely to translate to a cost-effective commercial manufacturing approach in the near term. Thus, strategies to reduce IgG1 N-glycan heterogeneity have the potential to improve the homogeneity of the product.

**Figure 1:**
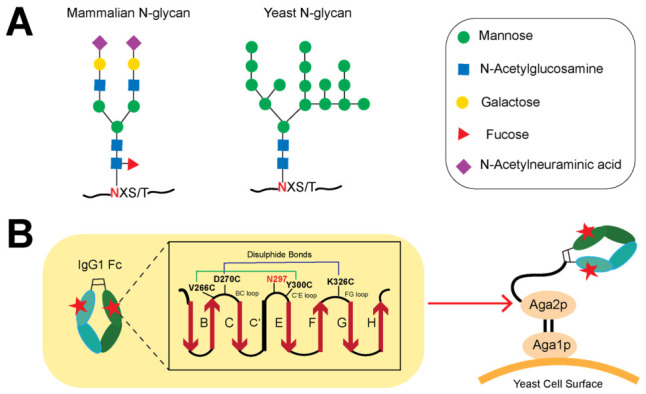
Two strategies to achieve IgG1 Fc compositional homogeneity. (**A**). Mammals produce proteins bearing highly remodeled N-glycans, whereas fungal expression platforms express proteins with high mannose glycoforms. (**B**). Stabilizing IgG1 Fc through disulfide bonds. Disulfide bonds linking residues V266C and Y300C (linking BC and C’E loops) and residues D270C and K326C (linking BC and FG loops) are shown in green and blue, respectively. Relative positions of the residues forming disulfide bonds are shown.

The impact of N-glycan composition on receptor binding also complicates engineering efforts because one common approach, using yeast surface display (YSD), fails to provide either appropriate mammalian N-glycan compositions or distributions of glycoforms. This shortcoming limits the applicability of glycosylated Fc fragments derived from YSD because the yeast-derived sequences may exhibit different properties with a mammalian N-glycan. In contrast to the mammalian expression platforms that produce therapeutic antibodies with highly heterogeneous and complex-type N-glycans, yeast synthesizes oligomannose-type N-glycans that may be extended to dozens of mannose residues (Figure 1A) [20,21]. Oligomannose-type glycoforms on yeast-expressed antibodies are rapidly cleared from the serum, greatly limiting the therapeutic window [22]. Thus, using yeast-based engineering platforms would optimize sequences using inappropriate glycans and is a particular concern considering the previous work showing specific interactions between mammalian N-glycan termini and Fc residues that promote receptor-binding affinity [11,23]. Yeasts expressing mammalian processing enzymes are available [24,25,26,27]. However, most of these strains were generated using Komagataella pastoris (Pichia) rather than Saccharomyces cerevisiae, the common YSD host. Furthermore, these strains produce variable levels of complex type N-glycans [21,24,25,26,28,29]. *S. cerevisiae* is considered superior for YSD due to efficient homologous recombination and the ability to support larger library sizes than Pichia [30].

We evaluated a *S. cerevisiae*-based YSD strategy to improve antibody compositional homogeneity. Several groups have previously developed aglycosylated variants with enhanced affinity for the FcγRIIIa receptor [31,32,33]. However, these efforts have not specifically incorporated the results of antibody structure/function studies that demonstrate the importance of the N-glycan in stabilizing loops that form the receptor-binding interface. In this work, we incorporated these structural insights to engineer aglycosylated Fc domains by introducing disulfide bonds designed to stabilize the FcγRIIIa-binding interface (Figure 1B).

## 2. Materials and Methods

### 2.1. Yeast Strain and Media

All reagents were purchased from Millipore Sigma unless stated otherwise. The yeast surface display strain, EBY100 [ATCC-MYA4941] (GAL1-AGA1:URA3 ura3-52 trp1 leu2Δ1 his3 Δ200 pep4: HIS2 prb1 Δ1.6R can1 GAL), that was used to create the Fc disulfide variants and yeast display libraries, was purchased from ATCC. Prior to transformation, EBY100 cells were grown in YPD medium (Millipore Sigma Y1375, St. Louis, MO, USA). Yeast transformed with the yeast display vector pYD1 (Agilent, Santa Clara, CA, USA) were plated on Minimal Dextrose plates (0.67 g/L Yeast Nitrogen Base without Amino Acids (Sigma Y0626), 0.1 mg/L Leucine and 2% Glucose). Yeast Surface Display Libraries were grown/induced in YNB-CAA [6.7 g/L Yeast Nitrogen Base Without Amino Acids (Sigma Y0626) and 0.5 g/L Casamino acids (Millipore Sigma CAS 65072-00-6)] with 2% glucose/galactose). Yeast Display Libraries generated using this strain were grown/induced in YNB-Dropout with 2% glucose/galactose.

### 2.2. Generate Fc Disulfide Variants and Surface Expression Characterization Through Flow Cytometry

Mutations corresponding to the three aglycosylated Fc disulfide variants described in the text (Fc T299A/D270C/K326C, Fc T299A/V266C/Y300C and Fc T299A/D270C/K326C/V266C/Y300C) were generated through Site-Directed Mutagenesis (SDM) by introducing the desired mutations within the primers and combining DNA fragments through fusion PCR [34]. The PCR fragment corresponding to the Fc domain was cloned into the yeast display vector pYD1 using the Nhe1 and EcoR1 restriction sites. Sequences for each of the generated constructs were validated through Sanger Sequencing. Each of these constructs was transformed into EBY100 cells through a standard Li-Ac yeast transformation protocol [35]. A positive transformant was grown overnight at 30 °C in YNB-CAA + 2% glucose and then induced at an OD_600_ = 0.5 in YNB-CAA + 2% galactose.

To determine the Fc surface expression of each of these variants, ~10^6^ induced yeast cells (24 h induction) were centrifuged at 1500× *g* for 1 min to remove the supernatant. The yeast pellet was then washed with 100 μL of 1× PBS, followed by a wash with 100 μL 1× PBSA (1× PBS + 1% BSA). The cells were then incubated with Rabbit Anti-hIgG (Primary Antibody) at 4°C for an hour. The cells were then washed with 100 μL of 1× PBSA and then incubated with PE Anti-Rabbit (Secondary Antibody) at 4 °C for another hour. These samples were run on FACSCanto (BD Biosciences, San Jose, CA, USA) to determine the surface expressions of the three individual Fc disulfide variants.

### 2.3. Creation of Yeast Surface Display (YSD) Libraries

To generate yeast surface display libraries, we amplified separate fragments of the pYD1 vector and assembled them in yeast through homologous recombination. Separate DNA fragments of the pYD1 vector backbone containing the selection marker (TRP1) and the CEN-ARS element were amplified using Pfu Turbo. The diversified Fc region (BC-FG loop) was amplified using the low-fidelity polymerase Taq Polymerase with low concentrations of the original template, along with 5 mM MgCl_2_ and 0.5 mM MnCl_2_ to decrease fidelity. All the DNA fragments described were gel-extracted using a Qiagen Gel Extraction Kit. These DNA fragments were then transformed into EBY100 with the Li-Ac yeast transformation protocol [35]. The transformed yeast cells were then plated onto the appropriate selection plate to recover the generated yeast display library.

### 2.4. Sorting and Diversifying YSD Libraries

Yeast cells containing the YSD library were grown overnight in YNB-CAA + 2% overnight at 30 °C. Cell density (OD_600_) was measured, and a cell culture volume corresponding to 5 × 10^6^ of cells was centrifuged at 3000× g for 10 min. The spent medium was removed, and the cells were resuspended in fresh induction medium (YNB-CAA) containing 2% galactose. The yeast culture was then induced for 24 h at 30 °C. For sorting, 10^7^ cells were centrifuged at 1500 g for 1 min and washed with 100 μL of 1× PBS, followed by a wash with a 100 μL 1× PBSA (1× PBS + 1% BSA). The yeast cell pellet was then stained with 363 nM Protein A-AF647 (Thermo Fisher, Waltham, MA, USA) and 2 μM GFP-FcγRIIIa at 4 °C for 1 h. After staining, the cells were then washed with 100 μL of 1× PBS twice. In the initial round of sorting, ~2% of the top GFP^+^, AF647^+^ cells were selected and isolated through gating. The sorted cells were then grown in ~1 mL of the appropriate selection medium at 30 °C for 2–3 days. This culture was then used for plasmid isolation using a Zymoprep kit (Zyme Research, Irvine, CA, USA). The plasmid isolated from the sorted cells from the first round was used as a template to generate a yeast display library for the next round. In the subsequent rounds of sorting, 0.1% of the top GFP^+^ and AF647^+^ cells were selected and isolated through gating. This process of diversification was repeated until we no longer saw a percentage improvement in the double-positive cells. The enriched library was diluted and plated on the appropriate selection plate to isolate individual colonies. Each of the individual colonies was inoculated into a 96 well containing selection medium and sent to Genewiz for Sanger Sequencing.

### 2.5. Expression and Purification of Selected Clones as hIgG1 Fc Domains

Gene-fragment coding for the Fc domains for the selected yeast surface display clones A-38, A-58, B-36, B-54, and B-57 were synthesized by Integrated DNA Technologies (IDT). Each of these gene fragments was cloned into the expression vector pGEN2 using restriction sites EcoRI and BamHI to generate an 8× HIS-GFP-TEV-Fc construct. Each ligation product was transformed into competent DH5α cells to recover positive transformants. The sequences for each of the clones were validated through Sanger Sequencing. A megaprep kit (Qiagen, Velmo, The Netherlands) was used to generate sufficient amounts of DNA to transfect HEK293F cells as previously described [36]. On day 5 post-transfection, the cell cultures were harvested by centrifugation at 14,000 rpm for 10 min. The supernatant was collected and diluted 1:1 by volume with 50 mM 3-(N Morpholino) propane sulphonic acid (MOPS), 200 mM potassium chloride, and 25 mM imidazole, pH 7.2, and applied to a Ni-NTA column containing 1 mL Protein A-Sepharose to isolate the Fc domain equilibrated with the same buffer. The column was washed with the same buffer containing 50 mM imidazole, then eluted with the same buffer containing 250 mM imidazole. The fractions containing the GFP-Fc variants were then pooled for further characterization; 10 µl of each fraction were diluted 1:1 with SDS Sample buffer to run on an SDS-PAGE gel and stained with Coomassie to determine the purity of the isolated antibody. The fractions containing IgG1 Fc domains were pooled and concentrated using an centrifugal concentrator filter (10 kDa cutoff, Millipore Sigma) and exchanged into a buffer containing 20 mM MOPS and 100 mM potassium chloride, pH 7.2. The concentration of the purified antibody was determined by measuring A280.

### 2.6. Surface Plasmon Resonance

IgG1 Fc was immobilized onto a CM5 chip (Cytiva, Marlborough, MA, USA), following as previously described [23]. Affinity measurements were carried out using a BiaCore T200 instrument (Cytiva). Increasing concentrations of GFP-FcγRIIIa were applied across the chip surface until equilibrium was reached. Affinity constants were determined by fitting the response curve using the T200 system software after double normalization (first to the surface-deactivated reference channel and then to a sample with zero concentration collected using the lane of interest). The response curve was fit to a 1:1 binding model. A data point corresponding to each concentration was collected by averaging within a 5 s window before equilibrium was reached. Reported errors indicate errors in the curve fitting procedure.

### 2.7. Crystallization, Data Collection, and Refinement

The IgG1 Fc wt, T299A, V266C/Y300C and D270C K326C variants were expressed in HEK293F cells and purified using a Protein A column followed by an S200 size exclusion column, which was pre-equilibrated using 20 mM MOPS and 100 mM sodium chloride, pH 7.2. Fractions containing protein were identified using an SDS-PAGE protein gel. Fractions containing Fc were concentrated using an centrifugal concentrator filter (10 kDa cutoff, Millipore Sigma) to a concentration of ~8–10 mg/mL. Initial screens for crystals were carried out at 18 °C using the hanging drop method. IgG1 Fc D270C K326C crystals formed as thin cylindrical rods in 0.1M HEPES pH 7.5, 10% PEG 3350. Diffraction data was collected at the Advanced Photon Source at Argonne National Laboratory on beamline 23-IDD using a PILATUS detector (DECTRIS AG, Baden-Dättwil, Switzerland). The data was indexed, merged, and scaled using HKL-2000 (version v722). Iterative molecular replacement was performed using Phenix Phaser-MR (Lawrence Berkeley Laboratory, Berkely CA) and an IgG1 Fc protein model (PDB ID: 1fc1). Final refinement was performed using Refmac5 (MRC Laboratory of Molecular Biology, Cambridge, UK) with isotropic B values.

## 3. Results

### 3.1. Engineering IgG1 Fc with Stabilizing Disulfide Bonds

**Figure 2:**
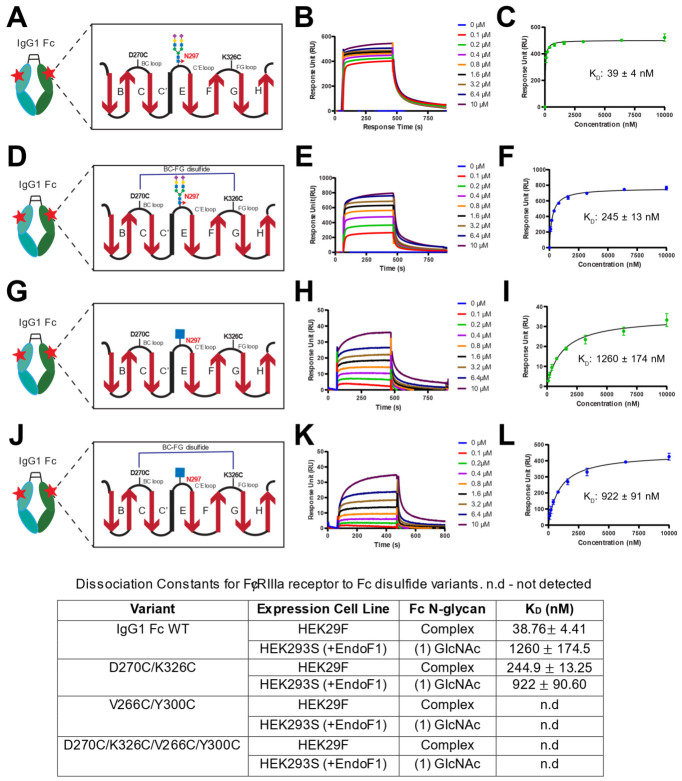
Affinity of the FcγRIIIa complex with IgG1 Fc determined with SPR. (**A**,**D**,**G**,**J**) Schematics depicting the location of the Fc N-glycan (complex type or (1) GlcNAc) with respect to the loops within WT Fc domain or Fc D270C/K326C variant, respectively. (**B**,**E**,**H**,**K**) Sensograms representing FcγRIIIa receptor binding with either wildtype Fc domain or Fc 270C/K326C, respectively. (**C**,**F**,**I**,**L**) Equilibrium binding curves for each of the binding interactions. Bottom, dissociation constants for the binding interactions between the Fc disulfide variants and FcγRIIIa. n.d.—not detected.

The IgG1 Fc N-glycan has been shown to stabilize the C’E loop that contributes to the FcγRIIIa-binding interface [11]. Eliminating the Fc N-glycan destabilizes this loop, reducing receptor-binding affinity. In addition, our group previously identified non-covalent interactions between the neighboring BC, C’E and FG loops that promote receptor-binding affinity [37]. Based on these findings, we created Fc variants containing disulfides in positions that are expected to stabilize the receptor-binding loops. We identified two bond locations connecting the BC to the FG loop and the BC to the C’E loop using the software Disulfide by Design v. 2.0 [38]: D270C/K326C and V266C/Y300C, respectively. We expressed three IgG1 Fc variants with D270C/K326C, V266C/Y300C, or D270C/K326C/V266C/Y300C mutations in HEK293S cells to compare the binding affinity to wildtype IgG1 Fc. HEK293S cells express proteins with oligomannose (Man5) N-glycans that can be trimmed to a minimal (1) GlcNAc N-glycan with EndoF1 following protein purification (Figure S1). This N-glycan remodeling strategy minimizes contacts from the majority of the N-glycan. Comparable to prior results, the glycosylated wildtype Fc variant (bearing a single GlcNAc) bound with a dissociation constant of 1.3 µM [11,37,39]. The glycosylated IgG1 Fc D270C/K326C variant (also bearing a single GlcNAc) bound with a slightly higher affinity of 0.9 µM, indicating that the mutations did not disrupt and may have in fact enhanced affinity (Figure 2). Neither the V266C/Y300C or the D270C/K326C/V266C/Y300C bound with a measurable affinity.

**Figure 3:**
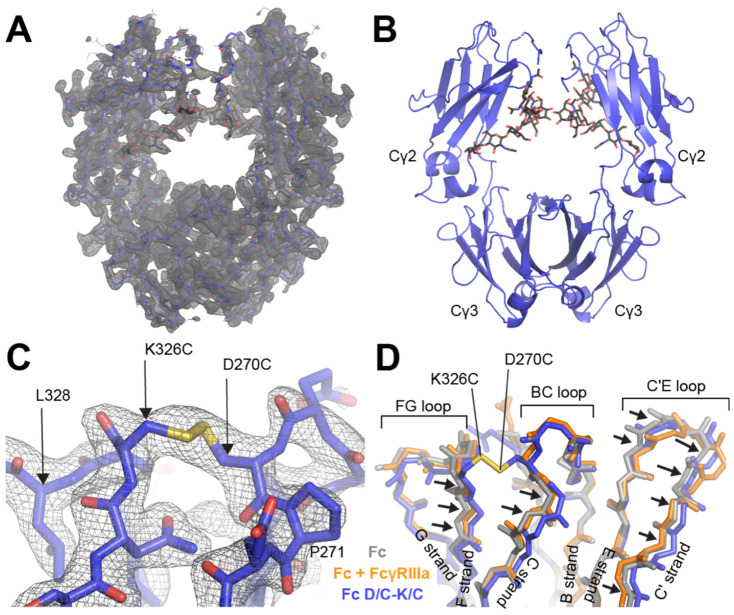
The IgG1 Fc D270C K326C variant forms a disulfide bond linking the FG and BC loops. (**A**). Electron density and (**B**). ribbon diagram showing the N-glycan in a black stick model of the Fc variant. (**C**). Close-up of the loop-linking disulfide bond. Electron density is scaled to 1.2 sigma for all maps. (**D**). Overlay of unliganded wt IgG1 Fc (grey sticks; pdb 4ku1) with IgG1 Fc with FcγRIIIa (orange sticks, pdb 5vu0) and IgG1 Fc D270C K326C (blue sticks; pdb 9bex).

A structure of the glycosylated IgG1 Fc D270C/K326C variant solved using X-ray crystallography at 2.25 Å resolution showed a global structure highly comparable to the wildtype (Figure 3; Table S1). We did not obtain crystals for the remaining two variants. A comparison of the similarity for the Cγ2 domains between this structure and that of the wildtype showed an rmsd of 0.485 Å (pdb 4ku1 [40]) and 0.351 Å when compared to IgG1 Fc in the IgG1 Fc:FcγRIIIa complex (pdb 5vu0 [39]). The electron density showed clear evidence supporting a disulfide bond linking D270C to K326C (Figure 3C). These mutations primarily affected the location of the C, C’ and F strands by shifting portions 1.0–1.8 Å, with minimal impact on the B, E and G strands. In addition to this, a comparison of the average B factors between the glycosylated IgG1 Fc D270C/K326C variant (pdb 9bex) and the previously published defucosylated wildtype Fc:FcγRIIIa complex (pdb 3ay4) [41] was made. We observed a significant difference in the average B factors between the BC, CʹE, and FG loops between the two structures, with a higher difference in the C’E loop between the two. This preliminary comparison provides some evidence that structural modifications made to the BC and FG loops in the Fc domain can reflect as structural changes in the C’E loop (Table S2).

### 3.2. YSD of Aglycosylated and Disulfide Bond-Stabilized IgG1 Fc Variants

**Figure 4:**
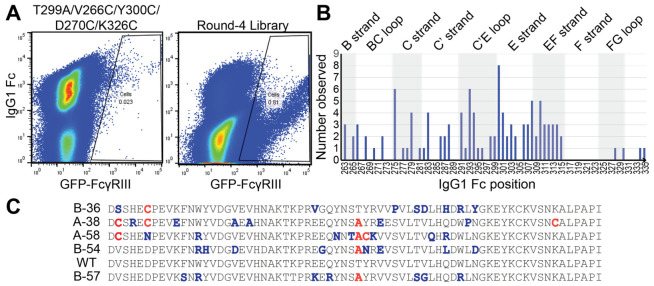
Selection of IgG1 Fc with novel disulfides for increased FcγRIIIa binding using yeast surface display libraries. (**A**) Library disulfide-A (**right**) was obtained after four rounds of sorting from the IgG1 Fc T299A/D270C/K326C/V266C/Y300C control (**left**). (**B**) Distribution of mutations in the clones derived from aglycosylated disulfide-A YSD library based on residue number. (**C**) Sequence alignment of aglycosylated clones derived from both disulfide-A (Clones 38 and 58) and disulfide-B libraries (Clones 36, 53 and 57) compared to the Fc wildtype sequence. Sequences from the resulting libraries were aligned using Clustal Omega (version 1.2.4). Mutations distinguishing the sequences of the library clones from the wildtype control are shown in blue, whereas starting cysteine and alanine mutations are shown in red.

This successful incorporation of disulfides into the receptor-binding loops on IgG1 Fc demonstrates that the cysteine modifications supported proper IgG1 Fc folding and expression and, in the case of the D270C/K326C, supported receptor binding. This success provides a foundation for circumventing N-glycosylation to achieve compositional homogeneity in a stabilized IgG Fc variant. We evaluated the ability of YSD to improve antibody-binding affinity for these aglycosylated disulfide-bonded variants. We observed the greatest enrichment in the Fc T299A/D270C/K326C/V266C/Y300C variant-derived library after four rounds of sorting with a 40-fold increase in IgG1 Fc-expressing (AF647+) clones that bound FcγRIIIa (GFP^+^) compared to our control (Figure 4A and Figure S2 and Table S3). DNA sequencing revealed that the majority of these clones were full-length and aglycosylated (94%). Of these, 49% retained cysteine residues from the original template with the potential to form at least one disulfide bond. Most of these Fc clones retained opposing cysteine residues poised to connect the BC and C’E loop (85%). Fewer clones retained cysteines to form a disulfide bond between the BC and FG loop (15%). We did not recover any Fc clones with all four loop cysteines from the original template. In each of these aglycosylated clones, we observed a distribution of mutations throughout the Fc domain, with more present in the C’E loop and adjoining regions (Figure 4B). This trend is consistent with previously discovered aglycosylated Fc variants showing enhanced FcγRIIIa engagement [33]. Additionally, we also observed a number of mutations localized in the BC loop; this result is in contrast to the prior observations showing that mutations were typically found in the C’E or FG loop in previous attempts to engineer aglycosylated Fc variants [31,32,33].

**Figure 5:**
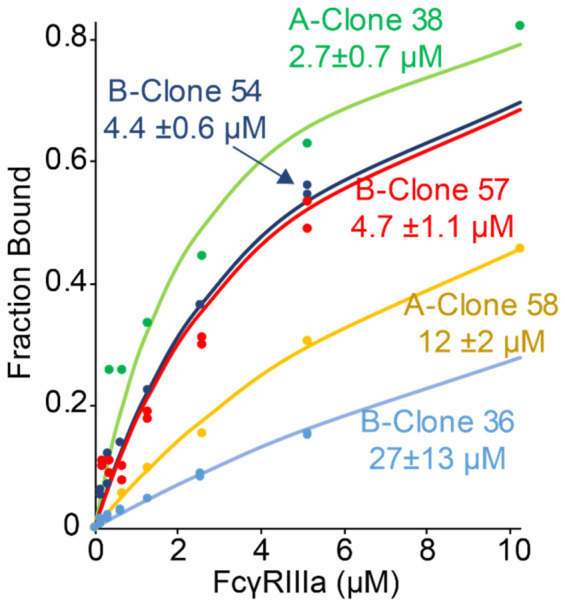
Affinity of selected IgG1 Fc clones from yeast surface display. These proteins were expressed as IgG1 Fc and evaluated for binding to FcγRIIIa using SPR. Most clones are aglycosylated (clone 36 is the exception). Clones 38 and 58 are from the DisulfideA library and contain BC-FG and BC-C’E disulfides, respectively. Remaining clones come from the DisulfideB library. Notably, the aglycosylated T299A control shows no binding to FcγRIIIa under these conditions. Points represent equilibrium values measured for each interaction, and lines show the best fit of a binding isotherm. Errors represent curve fitting errors.

The sequencing results demonstrated a high degree of variability across the library. To identify the tightest binding variants, we increased the stringency by sorting our YSD disulfide A library with lower GFP-FcγRIIIa concentrations in each round, followed by screening for Fc surface expression to generate the disulfide B library (Figure S3 and Figure 4 and Table S4). DNA sequencing revealed a higher degree of similarity in this library, with three main families of clones. We selected five clones from both libraries, representing discrete families of clones that are distinct from the wildtype to evaluate the binding affinity in vitro. Two variants selected for further analysis from the DisulfideA library contain cysteine residues poised to form disulfide bonds (clones A38 and A58). Three others selected from the DisulfideB library include clones B36, B54 and B57. Three of these clones are aglycosylated (A38, B54 and B57) while two contain glycan sites reintroduced during mutagenesis and selection (A58 and B36). Each variant expressed in mammalian HEK293F cells at a high level as a GFP fusion and showed a high degree of purity following TEV cleavage (Figure S5). Each variant demonstrated increased binding affinity when compared to the aglycosylated T299A control, which shows no binding [31,32,33]. Clone A38 showed the greatest affinity of 2.8 µM (Figure 5). Notably, clone A38 retained residues to form the BC-FG disulfide observed by X-ray crystallography. Also note that the clones were not treated to modify any potential reintroduced glycan prior to analysis.

## 4. Discussion

Previous engineering attempts mostly identified aglycosylated clones by chance; this strategy intentionally stabilizes key Fc features that improve the Fc-FcγRIIIa interaction to serve as a starting point for selectin aglycosylated variants with YSD [31,32,33]. Alternative strategies have engineered the Fc domain while retaining a full-length N-glycan [42,43], but suffer from the inability of yeast to make mammalian glycans suitable for YSD. Here, we describe a novel approach, applying our findings from structural studies of mouse IgG2 to develop aglycosylated Fc clones that bind FcγRIIIa [37]. In this rational design approach, we engineered disulfide bonds between the loops of the IgG1 Fc domain to compensate for the lack of N-glycan stabilization.

Analysis of the library indicated that the disulfides were not universally conserved, which is not surprising for two reasons. First, mutations disrupting the cysteine residues are likely present in the library at high frequency. Second, our mutagenesis and selection strategy did not specifically retain the cysteine residues, thus identifying any clones retaining the key cysteine residues in the libraries following multiple rounds of sorting as evidence supporting the potential utility of disulfide-bonded IgG1 Fc, in terms of compositional homogeneity and tight FcγR binding. Additionally, engineering disulfide bonds within the Fc domains has also been found to confer advantages such as higher thermostability [44,45].

Attempts to express the aglycosylated A38 and B54 Fc variants as full-length IgG1 molecules revealed low expression yields and failed to provide sufficient material to evaluate the results of the YSD yeast surface display in antibody-dependent cell-activation studies. Though the factors that limited expression remain undefined, it is clear that introducing disulfides into the Fc effector binding loops supports an ability to recover clones with measurable receptor-binding affinity. Thus, these data demonstrate that introducing disulfide bonds into the effector binding loops of an aglycosylated IgG1 Fc variant can compensate for the N-glycan-induced stabilization and receptor-binding affinity while preventing compositional homogeneity originating from the N-glycan.

## Data Availability

The original data presented in the study are openly available in the RCSB Protein Data Bank at [https://www.rcsb.org/structure/9BEX] (accessed on 30 April 2026).

## Supplementary Materials

The following supporting information can be downloaded at https://www.mdpi.com/article/10.3390/antib15040055/s1, Figure S1: Purity and TEV cleavage of the disulfide variants; Figure S2: Disulfide A library dendrogram shows the relationship of clones recovered from the YSD library that started with the T299A/D270C/K326C/V266C/Y300C IgG1 Fc variant; Figure S3: Selection of clones within the DisulfideA library with increased stringency to generate the DisulfideB library; Figure S4: DisulfideB library: Dendrogram represents clones recovered from the DisulfideA library following panning to select for clones with the highest affinity; Figure S5: Purification of each IgG1 Fc variant following expression and TEV cleavage to remove GFP; Table S1: Data collection and refinement statistics; Table S2: Comparison of Average B-factors between defucosylated Fc (PDB ID: 3AY4)41 and Fc D270C/K326C variant (PDB: 9BEX); Table S3: DisulfideA library: List of clones and their protein sequences recovered from the T299A/D270C/K326C/V266C/Y300C Fc YSD library; Table S4: DisulfideB library: List of clones and their protein sequences recovered from the DisulfideA YSD library following panning.
