# Supplementary material for: Aglycosylated Immunoglobulin G1 Fc Stabilized Through Disulfide Bond Addition Exhibits Compositional Homogeneity and Retains Fc γ Receptor IIIa/CD16a Binding
Source: Antibodies (Basel). 2026 Jun 25;15(4):55. doi: 10.3390/antib15040055 (PMC13398071; doi:10.3390/antib15040055)
Supplement: Supplementary file 1 [file antibodies-15-00055-s001.zip › antibodies-4324593-supplementary.pdf]

Supplemental materials for:

Aglycosylated immunoglobin G1 Fc stabilized through disulfide bond addition exhibits compositional homogeneity and retains Fc γ receptor IIIa / CD16a binding

Anjali Shenoy[1], Daniel J. Falconer[2], Adam W. Barb[1,3,4*]

Supplement Table S1. Data collection and refinement statistics

Wavelength (Å)	1.000
Resolution range	29.02 - 2.25 (2.34 - 2.25)*
Space group	P 21 21 21
Unit cell (a b c; Å)	50.023 79.74 142.478
(α β γ;°)	90 90 90
Total reflections	103598 (3716)
Unique reflections	46261 (2578)
Multiplicity	2.2 (1.4)
Completeness (%)	88.49 (52.30)
Mean I/sigma(I)	9.27 (1.11)
Wilson B-factor	47.68
R-merge	0.07876 (0.445)
R-meas	0.101 (0.5977)
R-pim	0.06231 (0.3953)
CC1/2	0.993 (0.641)
CC*	0.998 (0.884)
Reflections used in refinement	24614 (1589)
Reflections used for R-free	1213 (71)
R-work	0.2438
R-free	0.3039
Number of non-hydrogen atoms	3692
macromolecules	3373
ligands	227
solvent	92
Protein residues	430
RMS(bonds)	0.015
RMS(angles)	1.91
Ramachandran favored (%)	98.11
Ramachandran allowed (%)	1.89
Ramachandran outliers (%)	0
Rotamer outliers (%)	2.86
Clashscore	7.82
Average B-factor	51.79
macromolecules	50.88
ligands	70.14
solvent	39.79

* Values in parentheses reflect the outer shell

Supplementary Table S2. Comparison of Average B-factors between defucosylated Fc (PDB ID: 3AY4)[41] and Fc D270C/K326C variant (PDB: 9BEX)

Residue Positions	Secondary Structural Element	Average B-factor for PBD structure 3AY4	Average B-factor for PDB structure 9BEX
263-265	B strand	29.50	59.83
266-274	BC loop	38.66	83.31
275-282	C strand	57.88	54.66
283-290	C' strand	58.32	64.54
291-300	C'E loop	39.94	91.48
301-308	E strand	41.70	53.69
308-316	EF strand	48.35	47.53
316-324	F strand	51.07	53.53
325-330	FG loop	49.03	77.02

Residue Number	Amino Acid Residue	Average B factor (PDB ID: 3AY4)	Average B factor (PBD ID: 9BEX)
263	VAL	30.83	53.1
264	VAL	28.32	60.1
265	ASP	29.34	66.29
266	VAL	29.97	67.46
267	SER	32.09	74.9
268	HIS	32.44	85.99
269	GLU	38.33	99.05
270	ASP	40.71	102.89
271	PRO	36.88	91.02
272	GLU	48.5	92.14
273	VAL	39.47	68.1
274	LYS	49.51	68.26
275	PHE	40.85	49.67
276	ASN	55.39	55.62
277	TRP	52.8	52.67
278	TYR	66.41	55.63
279	VAL	61.66	53.01
280	ASP	66.21	55.98
281	GLY	61.82	60.06
282	VAL	63.38	71.76
283	GLU	62.81	65.71
284	VAL	60.09	59.36
285	HIS	61.62	67.67
286	ASN	58.58	61.86
287	ALA	51.85	58.44

288	LYS	57.03	57.64
289	THR	52.09	60.61
290	LYS	57.4	77.78
291	PRO	48.09	85.33
292	ARG	46.25	76.42
293	GLU	50.03	97.78
294	GLU	45.27	86.71
295	GLN	33.58	81.63
296	TYR	39.1	102.06
297	ASN	32.48	94.33
298	SER	35.61	95.94
299	THR	31.44	86.7
300	TYR	37.55	107.89
301	ARG	35.28	81.89
302	VAL	36.24	62.46
303	VAL	39.64	48.44
304	SER	40.8	43.18
305	VAL	44.45	43.84
306	LEU	48.09	46.87
307	THR	47.41	49.15
308	VAL	48.97	42.01
309	LEU	49.15	52.97
310	HIS	42.62	48.28
311	GLN	51.68	62.01
312	ASP	51.25	48.46
313	TRP	46.57	38.1
314	LEU	44.47	41.18
315	ASN	52.06	47.25
316	GLY	48.85	46.17
317	LYS	55.84	52.3
318	GLU	61.37	64.01
319	TYR	51.77	40.08
320	LYS	57.33	55.47
321	CYS	48.9	49.58
322	LYS	48.98	61.41
323	VAL	43.43	52.58
324	SER	43.2	60.15
325	ASN	39.79	61.92
326	LYS	50.64	88.12
327	ALA	45.65	67.17
328	LEU	48.37	78.89
329	PRO	55.03	85.14
330	ALA	56.3	80.89

Supplementary Table S3. DisulfideA library: List of clones and their protein sequences recovered from the T299A/D270C/K326C/V266C/Y300C Fc YSD library. These sequences support the dendrogram in Supplementary Fig 1.

**Wildtype**	CVVVDVSHEDPEVKFNWYVDGVEVHNAKTKPREEQYNSTYRVVSVLTVLHQDWLNGKEYKCKVSNKALPAPIEKTISK
**Clone-3**	CVVVDCNHECPEVKFNWCVDGVEVHNAKTKPREEQYNPACRAVSGLTALPQDWLTGKEYKCKVSNKALPAPIEKTISK
**Clone-8**	MRRSGCGHEDPEVEFNWYVDGVEVHDAKTKPREEQYNSACRAVSVLADLHQGWLNGKEYKCKVSNKALPAPIEKTISK
**Clone-11**	CVVVGCSHECPEVKLNRCVDGVEDHSAKTRPREEQCNSACKVVSVLTVQHRDWLNGKEYKCKVSNKALPAPIEKTISK
**Clone-13**	MRRSGCEHEDPEVKFNWYADGVEAHNAKTKPREGRYNSACRVVSVLTVLHQDWLNGKEYKCKVSNKALPAPIEKTISK
**Clone-16**	CVVLXCSHECPEVKFNWNVDGVEVHDAKTKPREEQYNSACRVVSVLTGLNLDWLNGEEYKCKVSNKALPAPIEKTISK
**Clone-33**	CVVVVAATKTRKSSLTGALTGMKITMQKTKPREGQYNSACRVVSGLTVLHQDWLNGKEYKCKVSNKALPAPIEKTISK
**Clone-35**	GCSHECPEVKLNRCVDGVEDHSAKTRPREEQCNSACKVVSVLTVQHRDWLNGKEYKCKVSNKALPAPIEKTISK
**Clone-37**	CVVVDVSRGCPEVKFNWYADGVEVHNAKTKPREGQYNSACRVVSVLTVLHQDWLNGKEYKCKVSNKALPAPIEKTISK
**Clone-38**	CVVVDCSRECPEVEFNWYVDGAEAHNAKTKPREEQYNSAYREESVLTVLHQDWPNGKEYKCKVSNCALPAPIEKTISK
**Clone-42**	MRRVDCSHEDPEVKFNWYDDGVEVHNAKTKPREELYYSACRVVPVLTVLHQDWLNGEEYKRKVSNKALPAPIEKTISK
**Clone-49**	CVVVDCSHECPEGKFNWYVDGVEVHNAKTKPREEQYNSACRLVSVLTVLHQDWLNGKEYKCKVSNCALPAPIEKTISK
**Clone-50**	MRRSGCEHEDPEVKFNRFVDGVEVHNAEEKPREEQYHSACSVVSVLPVLHQDWLNGKEYKCKVSNKALPAPIEKTISK
**Clone-51**	MRRSGCSHEDPEVKFNWYVDGVEVHNAKTKPREEQYNSACTVVSVLTVLHRDWLNGKEYKCKVSNKAPPAPIEKTISK
**Clone-52**	SGCGHEDPEVEFNWYVDGVEVHDAKTKPREEQYNSACRAVSVLADLHQGWLNGKEYKCKVSNKALPAPIEKTISK
**Clone-55**	CSHECPEVKFNRYIDGVEAHIAKTKPREEQYNSACRVVSVLTVLHQDWLNGKEYKCKVSNKALPAPIEKTISK
**Clone-57**	SGCSHEDPEVKFNWYVDGVEVHNAKTKPREEQYNSACTVVSVLTVLHRDWLNGKEYKCKVSNKAPPAPIEKTISKAK
**Clone-58**	CVVVDCSHENPEVKFNRYVDGVEVHNAKTKPREEQNNTACKVVSVLTVQHRDWLNGKEYKCKVSNKALPAPIEKTISK
**Clone-61**	CVVLDCSRECPEVEFNWYVDGAEAHNAKTKPREEQYNSAYREESVLTVLHQDWPNGKEYKCKVSNCALPAPIEKTISK
**Clone-62**	MRRSGCEHEDPEVKFNRFVDGVEVHNAEEKPREEQYHSACSVVSVLPVLHQDWLNGKEYKCKVSNKALPAPIEKTISK
**Clone-65**	CVVVDRSYECPEVKFNWCVDGVEVHNAKTKPREEQYNSACRVVSVLTVLHRDWLNGKEYKCKVSNKALPAPIEKTISK
**Clone-66**	CVVVDCNHECPEVKFNWCVDGVEVHNAKTKPREEQYNPACRAVSGLTALPQDWLTGKEYKCKVSNKALPAPIEKTISK
**Clone-68**	CVVVDCSHECPEVKFNWNVDGVEVHDAKTKPREEQYNSACRVVSVLTGLNLDWLNGEEYKCKVSNKALPAPIEKTISK
**Clone-70**	CVVVDRSYECPEVKFNWCVDGVEVHNAKTKPREEQYNSACRVVSVLTVLHRDWLNGKEYKCKVSNKALPAPIEKTISK
**Clone-73**	CVVVDVSHGCPEVRFNRYVDGAEVHNAKTRPREEQYNSACRVVSASLVVHQDSKNRKEYKDKLSSRANTAPVEQTK
**Clone-78**	MRRSGCGHEDPEVEFNWYVDGVEVHDAKTKPREEQYNSACRAVSVLADLHQGWLNGKEYKCKVSNKALPAPIEKTISK
**Clone-82**	CVVVDCSRECPEVEFNWYVDGAEAHNAKTKPREEQYNSAYREESVLTVLHQDWPNGKEYKCKVSNCALPAPIEKTISK
**Clone-84**	CVVVDCSHECPEGKFNWYVDGVEVHNAKTKPREEQYNSACRLVSVLTVLHQDWLNGKEYKCKVSNCALPAPIEKTISK
**Clone-86**	CVVVDRSYECPEVKFNWCVDGVEVHNAKTKPREEQYNSACRVVSVLTVLHRDWLNGKEYKCKVSNKALPAPIEKTISK

Supplementary Table S4. DisulfideB library: List of clones and their protein sequences recovered from the DisulfideA YSD library following panning. These sequences support the dendrogram in Supplementary Fig 3.

**Wildtype**	CVVVDVSHEDPEVKFNWYVDGVEVHNAKTKPREEQYNSTYRVVSVLTVLHQDWLNGKEYKCKVSNKALPAPIEKTISK
**Clone-6**	CVEVDVSHEDPEVKFNRHVDGDEVHDAKTKPREGQYNSANREVSVLTVLHLDWLDGKEYKCKVSNKALPAPIEKTISK
**Clone-8**	CVEVDVSHXNPEVKFNRHVDGDEVHDAKTKPREGQYNSANREVSVLTVLHLDWLDGKEYKCKVSNKALPAPIEKTISK
**Clone-10**	CVEVDVSHEDPEVKFNRHVDGDEVHDAKTKPREGQYNSANREVSVLTVLHLDWLDGKEYKCKVSNKALPAPIEKTISK
**Clone-13**	CVXXDVSHEDPEVKFNRHVDGDEVHDAKTKPREGQYNSANREVSVLTGLHLDWLDGKEYKCKVSNKALPAPIEKTISK
**Clone-14**	CVVLDVSHENPEVKSNRYVDGVEVHNAKTTPRKERYNSAYRVVSVLSGLHQDRLNGKEYKCKVSNKALPAPIEKTISK
**Clone-15**	CVVADSSHECPEVKFNWYVDGVEVHNAKTKPRVGQYNSTYRVVPVLSDLHHDRLYGKEYKCKVSNKALPAP
**Clone-16**	CVEVDVSHENPEVKFNRHVDGDEVHDAKTKPREGQYNSANREVSVLTVLHLDWLDGKEYKCKVSNKALPAPIEKTISK
**Clone-29**	PGNPEVKFNRHVDGDEVHDAKTKPREGQYNSANREVSVLTVLHLDWLDGKEYKCKVSNKALPAPIEKTISK
**Clone-31**	CVVVDVSHEDPEVKSNRYVDGVEVHNAKTTPRKERYNSAYRVVSVLSGLHQDRLNGKEYKCKVSNKALPAPIEKT
**Clone-32**	CVVVDVSHEDPEVKSNRYVDGVEVHNAKTTPRKERYNSAYRVVSVLSGLHQDRLNGKEYKCKVSNKALPAPIEKTISK
**Clone-33**	CVEVDVSHENPEVKFNRHVDGDEVHDAKTKPREGQYNSANREVSVLTVLHLDWLDGKEYKCKVSNKALPAPIEKTISK
**Clone-34**	DVSHEDPEVKFNRHVDGDEVHDAKTKPREGQYNSANREVSVLTVLHLDWLDGKEYKCKVSNKALPAPIEKTISK
**Clone-36**	CVVADSSHECPEVKFNWYVDGVEVHNAKTKPRVGQYNSTYRVVPVLSDLHHDRLYGKEYKCKVSNKALPAPIEKT
**Clone-38**	CVEVDVSHEDPEVKFNRHVDGDEVHDAKTKPREGQYNSANREVSVLTVLHLDWLDGKEYKCKVSNKALPAPIEKTISK
**Clone-40**	CVVADSSHECPEVKFNWYVDGVEVHNAKTKPRVGQYNSTYRVVPVLSDLHHDRLYGKEYKCKVSNKALPAPIEKT
**Clone-44**	CVEVDVSHEDPEVKFNRHVDGDEVHDAKTKPREGQYNSANREVSVLTVLHLDWLDGKEYKCKVSNKALPAPIEKTISK
**Clone-47**	CVEVDVSHENPEVKFNRHVDGDEVHDAKTKPREGQYNSANREVSVLTVLHLDWLDGKEYKCKVSNKALPAP
**Clone-50**	VDVSHEDPEVKFNRHVDGDEVHDAKTKPREGQYNSANREVSVLTVLHLDWLDGKEYKCKVSNKALPAPIEKTISK
**Clone-53**	VVADVSHEDPEVEFNWYVNGVEDHNAKTRPREEQYNSARSVVSDLTVPHQDWLNGKEYKCKLSNKALPAPIEKTISK
**Clone-54**	CVEVDVSHEDPEVKFNRHVDGDEVHDAKTKPREGQYNSANREVSVLTVLHLDWLDGKEYKCKVSNKALPAPIEKTISK
**Clone-55**	NMRRVDCSHEDPEVKFNWYDDGVEVHNAKTKPREELYYSACRVVPVLTVLHQDWLNGEEYKRKVSNKALPAPIEKTISK
**Clone-56**	CVVXDVXHENPEVKSNRYVDGVEVHNAKTTPRKERYNSAYRVVSVLSGLHQDRLNGKEYKCKVSNKALPAPIEKTISK
**Clone-57**	CVVVDVSHEDPEVKSNRYVDGVEVHNAKTTPRKERYNSAYRVVSVLSGLHQDRLNGKEYKCKVSNKALPAPIEKTISK
**Clone-64**	CVVPDGSPXSPEVKFNWYVDGVEVHNAKTKPRVGQYNSTYRVVPVLSDLHHDRLYGKEYKCKVSNKALPAPIEKTISK
**Clone-65**	CVEVDVSHEDPEVKFNRHVDGDEVHDAKTKPREGQYNSANREVSVLTVLHLDWLDGKEYKCKVSNKALPAPIEKTISK
**Clone-66**	CVVADSSHECPEVKFNWYVDGVEVHNAKTKPRVGQYNSTYRVVPVLSDLHHDRLYGKEYKCKVSNKALPAPIEKTISK
**Clone-68**	CVVPXXSXXSPEVKFNWYVDGVEVHNAKTKPRVGQYNSTYRVVPVLSDLHHDRLYGKEYKCKVSNKALPAPIEKTISK
**Clone-69**	CVEVDVSHEDPEVKFNRHVDGDEVHDAKTKPREGQYNSANREVSVLTVLHLDWLDGKEYKCKVSNKALPAPIEKTISK
**Clone-70**	CVEVDVSHEDPEVKFNRHVDGDEVHDAKTKPREGQYNSANREVSVLTVLHLDWLDGKEYKCKVSNKALPAP
**Clone-71**	HGVVADSSHECPEVKFNWYVDGVEVHNAKTKPRVGQYNSTYRVVPVLSDLHHDRLYGKEYKCKVSNKALPAPIEKTISK
**Clone-72**	CVEVDVSHEDPEVKFNRHVDGDEVHDAKTKPREGQYNSANREVSVLTVLHLDWLDGKEYKCKVSNKALPAPIEKTISK
**Clone-73**	CVEVDVSHEDPEVKFNRHVDGDEVHDAKTKPREGQYNSANREVSVLTVLHLDWLDGKEYKCKVSNKALPAPIEKTISK
**Clone-74**	CVEVDVSHEDPEVKFNRHVDGDEVHDAKTKPREGQYNSANREVSVLTVLHLDWLDGKEYKCKVSNKALPAPIEKTI
**Clone-75**	CVVADSSHECPEVKFNWYVDGVEVHNAKTKPRVGQYNSTYRVVPVLSDLHHDRLYGKEYKCKVSNKALPAPIEKTISK
**Clone-76**	CVEVDVSHEDPEVKFNRHVDGDEVHDAKTKPREGQYNSANREVSVLTVLHLDWLDGKEYKCKVSNKALPAPIEKTIXK
**Clone-77**	CVVADSSHECPEVKFNWYVDGVEVHNAKTKPRVGQYNSTYRVVPVLSDLHHDRLYGKEYKCKVSNKALPAPIEKTISK
**Clone-78**	CVVADSSHECPEVKFNWYVDGVEVHNAKTKPRVGQYNSTYRVVPVLSDLHHDRLYGKEYKCKVSNKALPAPIEK
**Clone-79**	CVEVDVSHEDPEVKFNRHVDGDEVHDAKTKPREGQYNSANREVSVLTVLHLDWLDGKEYKCKVSNKALPAPIEKTISK
**Clone-81**	CVEVDVSHEDPEVKFNRHVDGDEVHDAKTKPREGQYNSANREVSVLTVLHLDWLDGKEYKCKVSNKALPAPIEKTISK
**Clone-82**	CVEVDVSHEDPEVKFNRHVDGDEVHDAKTKPREGQYNSANREVSVLTVLHLDWLDGKEYKCKVSNKALPAPIEKTISK
**Clone-83**	CVVADSSHECPEVKFNWYVDGVEVHNAKTKPRVGQYNSTYRVVPVLSDLHHDRLYGKEYKCKVSNKALPAPIEKTISK
**Clone-84**	CVEVDVSHEDPEVKFNRHVDGDEVHDAKTKPREGQYNSANREVSVLTVLHLDWLDGKEYKCKVSNKALPAPIEKTISK
**Clone-85**	CVEVDVSHEDPEVKFNRHVDGDEVHDAKTKPREGQYNSANREVSVLTVLHLDWLDGKEYKCKVSNKALPAPIEKTISK
**Clone-86**	CVVADSSHECPEVKFNWYVDGVEVHNAKTKPRVGQYNSTYRVVPVLSDLHHDRLYGKEYKCKVSNKALPAPIEKTISK
**Clone-87**	CVEVDVSHEDPXVKFNRHVDGDEVHDAKTKPREGQYNSANREVSVLTVLHLDWLDGKEYKCKVSNKALPAPIEKTISK
**Clone-88**	CVEVDVSHEDPEVKFNRHVDGDEVHDAKTKPREGQYNSANREVSVLTVLHLDWLDGKEYKCKVSNKALPAPIEK
**Clone-89**	CXELFXSHENPEVKFNRHVDGDEVHDAKTKPREGQYNSANREVSVLTVLHLDWLDGKEYKCKVSNKALPAPIEKT
**Clone-90**	CVEVDVSHEDPEVKFNRHVDGDEVHDAKTKPREGQYNSANREVSVLTVLHLDWLDGKEYKCKVSNKALPAPIEKTISK
**Clone-91**	CVVADSSHECPEVKFNWYVDGVEVHNAKTKPRVGQYNSTYRVVPVLSDLHHDRLYGKEYKCKVSNKALPAPIEKTISK
**Clone-92**	CVEVDVSHEDPEVKFNRHVDGDEVHDAKTKPREGQYNSANREVSVLTVLHLDWLDGKEYKCKVSNKALPAPIEKTISK

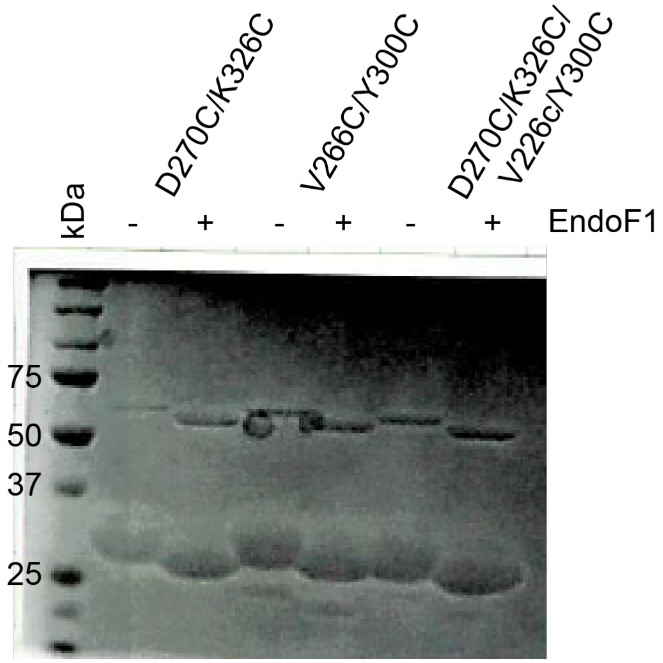

Supplementary Figure S1. Purity and TEV cleavage of the disulfide variants.

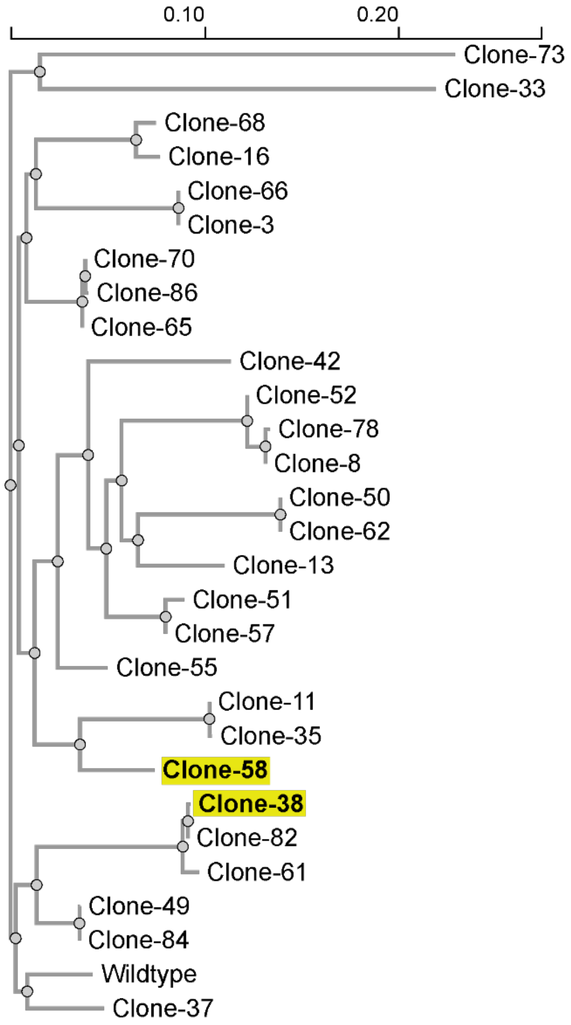

Supplementary Figure S2. Disulfide A library dendrogram shows the relationship of clones recovered from the YSD library that started with the T299A/D270C/K326C/V266C/Y300C IgG1 Fc variant. Horizontal lines in the dendrogram representing extent of sequence diversity (number of substitutions/lengths of protein sequence) can be determined using the scale at the top of the figure.

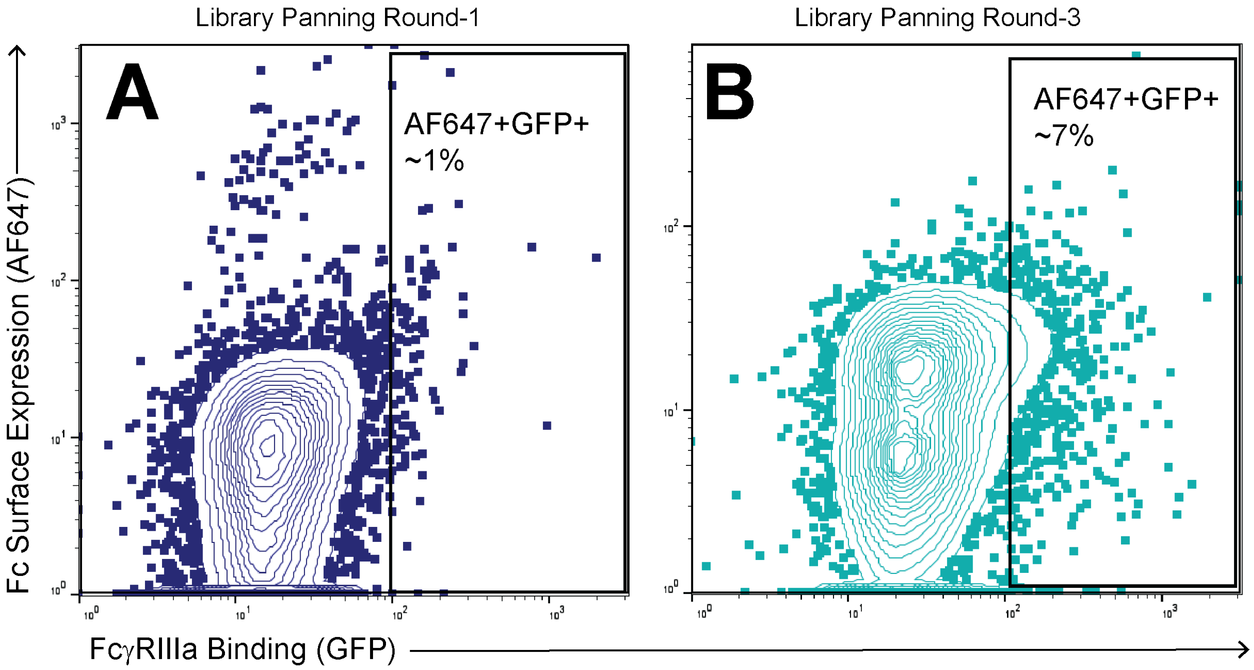

Supplementary Figure S3. Selection of clones within the DisulfideA library with increased stringency to generate the DisulfideB library. Enrichment in double positive cells (AF647^{+}GFP^{+}) from Round-1 (**A**) to Round-3 (**B**) was achieved.

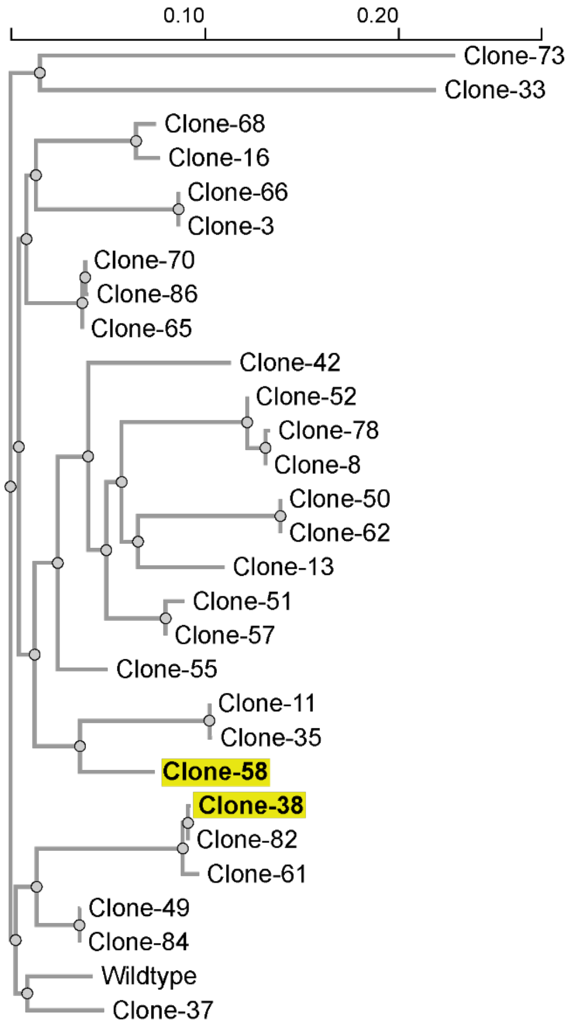

Supplementary Figure S4. DisulfideB library: Dendrogram represents clones recovered from the DisulfideA library following panning to select for clones with the highest affinity. Horizontal lines in the dendrogram representing extent of sequence diversity (number of substitutions/lengths of protein sequence) can be determined using the scale at the top of the figure.

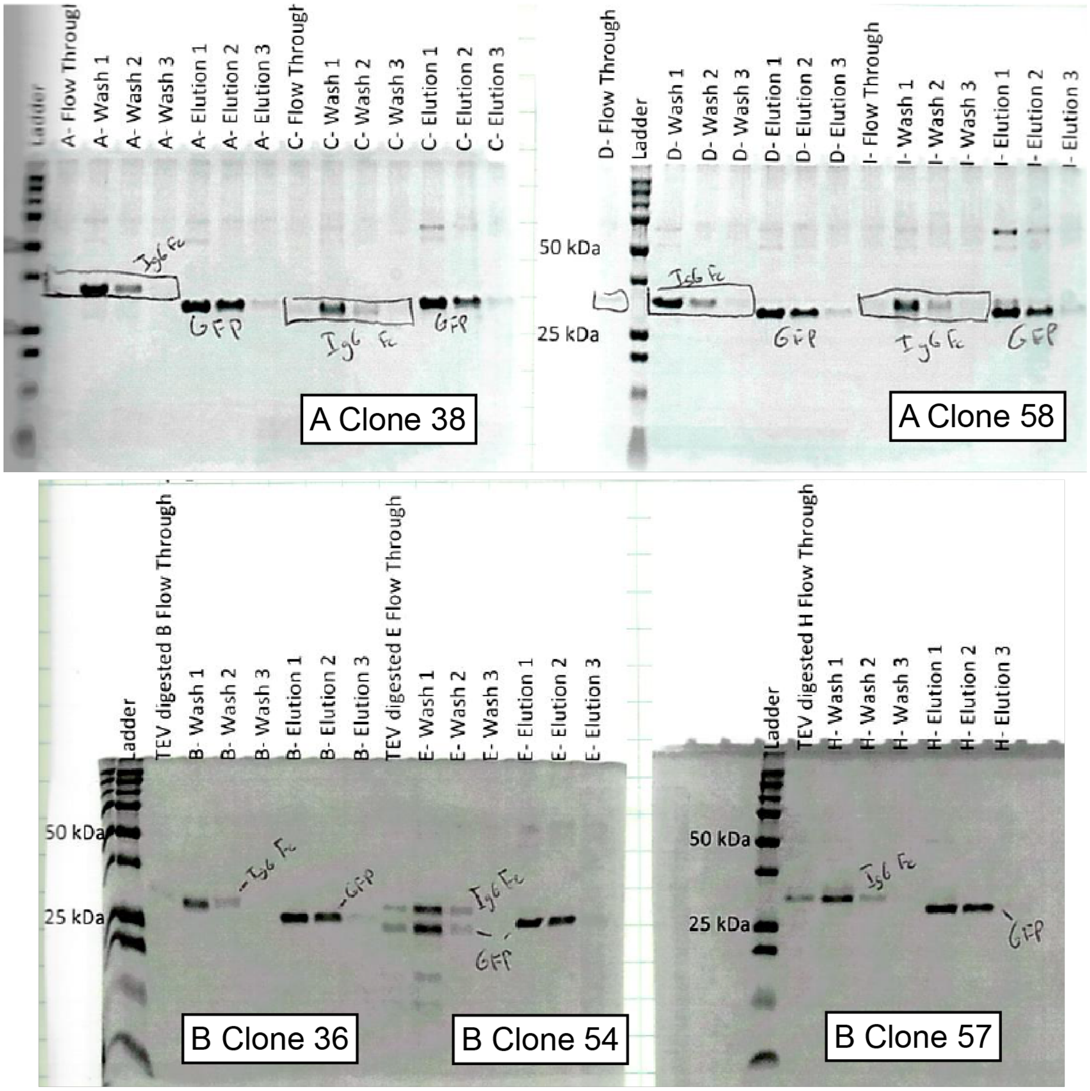

Supplementary Figure S5. Purification of each IgG1 Fc variant following expression and TEV cleavage to remove GFP.
